# Screening for Disease

**Published:** 1991-06

**Authors:** Petr Skrabanek

**Affiliations:** Department of Community Health, Trinity College, Dublin


### Screening for Disease - Possibilities and Problems

**Southmead Foundation Lecture delivered at Southmead Hospital Bristol November 1990***

**Petr Skrabanek**
Department of Community Health, Trinity College, Dublin

### INTRODUCTION

Towards the end of the last century the threat of tuberculosis (TB) led to mass TB detection programmes. The idea of screening for other diseases emerged around the same time and screening examination of healthy people was encouraged by the medical profession, factory owners, immigration authorities, health departments, and the army.

In the last two or three decades, further development has taken place in screening for cancer, prenatal screening, screening for risk factors for coronary heart disease, and multiphasic health checks (periodic health examinations). Leaving aside for the moment any evaluation of the evidence that such screening is of benefit to individuals as opposed to the various third parties, including the screeners themselves, it is important to examine the principles and criteria of screening.

### CRITERIA FOR SCREENING

The Principal Medical Officer of the British Ministry of Health, J.M.G. Wilson and the Swedish biochemist, G. Jungner wrote a key monograph on this subject[1], in which they stressed that screening tests should be validated before they are applied to populations; that the effect of screening must be evaluated in terms of reduced morbidity and mortality, and that early detection is unlikely to be cheaper than conventional curative medicine, "since more people will be found to be in need of treatment, and these will be mainly elderly persons liable to be under care for a long time''. The authors listed ten criteria for the rational assessment of screening.

1. *The condition sought should be an important health problem.* This is a relative concept. For example, within limited health resources, the state may not be able to afford both breast cancer screening and cervical cancer screening. In such case, breast cancer screening should have priority as it is more common than cervical cancer, and would save proportionally more lives. This assumes, of course, that such screening programmes do reduce mortality from the disease.
2. *There should be an accepted treatment for patients with recognised disease.* Unfortunately, this criterion is not fulfilled for any of the major cancers for which screening is advocated or practised. In fact it was the very lack of adequate treatment which pinned hopes (possibly false) on better results if the same treatment was applied earlier. In the case of prenatal screening, "treatment'' equals abortion.
3. *Facilities for diagnosis and treatment should be available.* Even the wealthiest countries find it difficult or impossible to finance and resource the increasing number of screening programmes thought to be feasible. The vacuum may be then filled by profit-making private organisations who offer screening facilities at a cost, but sometimes without adequate quality control.
4. *There should be a recognisable latent or early symptomatic stage.* This is not of itself a guarantee that the natural history of the disease can be altered. While all big tumours started as small tumours, it does not mean that clinically "small'' or "early'', is early in the biological sense.
5. *There should be a suitable test or examination.* This presumably means a test which is relatively simple to perform, sensitive and specific, and not too expensive. Commonly used cancer screening tests (for example, "Pap'' smear, mammography, Haemoccult) do not meet this criterion, as their predictive value is unacceptably low. This is a general problem which is discussed below.
6. *The test should be acceptable to the population.* A low acceptance rate, which decreases even further with subsequent recalls, is a common problem encountered in many screening programmes.
7. *The natural history of the condition should be adequately understood.* This criterion is not met for most cancers or for coronary heart disease.
8. *There should be an agreed policy on whom to treat as patients.* The literature on cervical cancer screening, for example, is replete with arguments and disagreements on what to do when various forms of "premalignant'' lesions are discovered. The same applies for uncertainity about the management of premalignant lesions in the breast, or of intestinal polyps. The cut-offs of "normal'' cholesterol or blood pressure are arbitrary.
9. *The cost of case-finding (including diagnosis and treatment of patients diagnosed) should be economically balanced in relation to possible expenditure on medical care as a whole.* "Case-finding'' in Wilson and Jungner's terminology refers to population screening as a preventive measure as opposed to screening as an epidemiological study. This criterion is rarely heeded as screening policies are decided mainly by political expediency.
10. *Case-finding should be a continuing process and not a once for all project.*

An important omission in the Wilson-Jungner list is an ethical criterion, which may be formulated as follows:

11. *Screening is only justified if the prospective screenees are fully informed about the potential risks and disadvantages as well as the benefits of screening.* The position of a doctor offering screening is quite different from that of a doctor who is responding to a patient's concerns. And even if the patient asks for screening, "his request is based on the belief that the procedure is of value, and if it is not, it is for the medical people to make this known''[2].

Similarly, Cochrane and Holland wrote: "We believe that there is an ethical difference between everyday medical practice and screening. If a patient asks a medical practitioner for help, the doctor does the best he can. He is not responsible for defects in medical knowledge. If, however, the practitioner initiates screening procedures, he is in a very different situation. He should, in our view, have conclusive evidence that screening can alter the natural history of disease in a significant proportion of those screened''[3].

Sackett and Holland introduced an ideological/political dimension into screening, drawing a distinction between the advocates of screening ("evangelists'') and the advocates of the

*Reprinted with permission from the Journal of Biomedical Sciences Vol. 1 No. 1 1990

scientific method ("snails")[4]. This conflict is apparent at present between those who advocate randomised controlled trials and those who have already been convinced that the available evidence is overwhelming and that the current randomised trials are unethical.

This difference was summarised by Sackett and Holland as follows:

"The advocates of screening, usually for impeccable motives, conclude that the pre-existing evidence plus commonsense — in the face of the ongoing toll of disability and untimely death — demand mass screening programme for the detection of citizens with the risk factors for these disorders now, even in the absence of experiments to determine whether the alteration of many risk factors will, in fact, alter risk. In "keeping the faith", screening advocates may find themselves forced to accept or reject evidence not so much on the basis of its scientific merit as on the extent to which it supports or rejects the stand that screening is good"[4].

### PREDICTIVE VALUE OF TESTS

The manufacturers of screening kits extol their wares by referring to the high specificity and sensitivity of the test. This often is a very misleading measure of the usefulness of the test for population screening. Consider the following example, used by a mathematician S.W. Golomb in an article in *The Los Angeles Times* (Dec 1988):

"The Z virus is present randomly in an average of one in 1000 chickens, the new SNAP test for the Z virus is 99% reliable — it misses only 1% of the infected chickens, and falsely identifies only 1% of the uninfected chickens as "positive". A chicken is selected at random and given the SNAP test. The result comes back positive. What is the probability that the chicken has the Z virus?"

It may surprise the reader that the result is not 99% but 9%. In other words, when the test comes back as "positive", 91 out of 100 such tests would be falsely positive. The performance of most screening tests actually used in humans are even less reliable that the SNAP test in the above example. When a battery of tests is used, for example in "executive health screening" on offer by various private clinics, the likelihood of some "positive" result becomes a near certainty. Large profits are made in this way even though evidence is lacking that multiphasic screening is of any benefit[5,6].

Screening for any particular disease presents specific theoretical and practical problems. Due to constraints on space three diseases for which population screening is widely advocated and practised have been chosen as examples.

### BREAST CANCER SCREENING

Since the first randomised controlled trial of mammography combined with physical examination in the USA in 1960s, three additional controlled trials have been carried out (the Kopparberg & Östergotland trial, the Malmö trial and the Edinburgh trial). Another larger trial is in progress in Canada. Published results are based on data derived from over 2 million woman-years observations, yet the evidence in favour of mammography is controversial[7,11].

For example, in the Malmö trial, which was a model of planning and execution, the observed benefit was so small that about 70,000 women would have to be offered screening for one death from breast cancer prevented or postponed annually.[12] Much more impressive results were reported in studies using the case-control design rather than a randomised controlled trial — breast cancer mortality was said to be reduced by 50% or more by screening centres in Holland (Nijmegen, Utrecht) and Italy (Florence). However, when Ranstam applied the case-control methodology retrospectively to the results from the Malmö trial he obtained a highly significant result of 58% reduction in breast cancer mortality![13]. Yet in this trial the real relative reduction in mortality from breast cancer was only 4%. Obviously, the case-control methodology as presently used is unsuitable and invalid for assessing breast screening benefits.

In the Edinburgh randomised controlled trial (14) a non-significant reduction in mortality was reported, and the director of the trial, Maureen Roberts, shortly before her own death from breast cancer, asked "are we brainwashing ourselves into thinking that we are making a dramatic impact on a serious disease before we brainwash the public?"[15].

As the benefits of mass mammography are questionable, the negative aspects of such screening assume even greater importance. Mammography is not a suitable screening test as it produces a large number of false positive results and misses up to 50% of true cancers. According to the latest Canadian results, the positive predictive value of mammography is less than 10%, that is, out of 100 "positive" mammograms less than 10 are truly positive. But even positive mammograms may create dilemmas of management.

No agreement exists on the treatment of various stages of breast cancer, particularly carcinomas *in situ*[17]. Modern mammography leads to overdiagnosis of lesions which otherwise would not be discovered and some would not progress to invasive stage within a lifetime. Furthermore, early diagnosis of breast cancer in most women is no guarantee of a cure but it extends the time the woman has to live with the knowledge of having an incurable disease, thus adding "extra cancer years" and additional anguish.

Schmidt calculated that for each woman who would benefit from mammography screening, 18 women would earn "extra cancer years" and 100 women would undergo unnecessary breast biopsy, including an unknown number of women with unnecessary mastectomy[18]. In Sweden, the number of breast operations doubled following the introduction of screening in one province.

### CERVICAL CANCER SCREENING

Cervical cancer is a relatively uncommon disease: in Ireland an average general practitioner would encounter one death from this disease every forty years. The "Pap" smear is an unsuitable screening test since its positive predictive value is about 1%. The test detects abnormalities which may or may not be precursors of invasive cancer, and there is no additional test with which this dilemma can be resolved. The interpretation of the test was described by the doyen of cervical cytology, Leopold Koss, as "the most difficult area of microscopy", and he warned that it is "singularly misleading" to attempt clarification of an atypical smear by obtaining a second smear[19]. Even experienced histopathologists show considerable interobserver variation in interpreting cervical abnormalities (20).

Giles *et al.* found about 10% of women screened systematically in a general practice (in a predominantly middle class area) positive on cytology and colposcopy, with a false negative rate of over 50%. Discovered abnormalities left the authors in a difficult position as they were unable, "cytologically, colposcopically, or histologically to predict which lesions will progress to invasive disease"[21].

Overtreatment then is inevitable. As there is no agreement on what treatment is appropriate for various "premalignant" lesions, many women may receive unnecessary treatment which can compromise their fertility or future pregnancy, while others may have unnecessary hysterectomy.

Yet we have no clear evidence that mass screening for cervical cancer affects mortality from the disease. In Britain, where some 4 million smears are taken annually, there has been no demonstrable impact on the trend in cervical cancer mortality. As pointed out in a *Lancet* editorial, "since cytological screening was introduced on a large scale around 1964, mortality has declined at 1% a year; but that seems to be the rate at which it has been falling for several decades previously. There was no obvious change." The editorialist also calculated that for every life saved by cervical screening, 40,000 smears and 200 excision biopsies were carried out -"a grievously poor cost-benefit ratio"[22].

It is ironic that criticism of cervical screening is received with greater hostility than criticism of breast cancer screening, as for breast cancer screening, as for breast cancer there are at least some randomised controlled trials suggesting that there may be a benefit to screened women, while for cervical cancer there are non. Critiques of cervical screening[23–27] have fallen on deaf ears.

### CHOLESTEROL SCREENING

Mass cholesterol screening is based on several assumptions: that the diet-heart hypothesis is true, that a reduction of blood cholesterol is achievable by a change in diet, and that the achieved reduction will reduce the mortality from coronary heart disease. The diet-heart hypothesis has been questioned by many critics[28–38]. Randomised controlled trials of cholesterol reduction by diet have failed to show any benefit. The most recent dietary trial from Minnesota Coronary Survey is particularly instructive[39]. In this study the compliance with the prescribed diet was achieved by enrolling 10,000 institutionalised men and women, half of whom were served an ordinary diet (saturated fat 18% of calories, polyunsaturated fat 5% of calories, P/S ratio 0.3, cholesterol 446 mg/day), and the other half an experimental diet (saturated fat 9% of calories, polyunsaturated fat 15% of calories, P/S ratio 1.6, cholesterol 166 mg/day). After 5 years of follow-up, the mean serum cholesterol in the experimental group fell by 15%, but the primary end-points (acute and silent infarction and sudden deaths) in this group were the same as in the controls, and the overall mortality in the experimental group was slightly higher.

When all randomised controlled trials of primary prevention of coronary heart disease were reviewed no benefit was demonstrable in participants. And yet some of these trials modified not only cholesterol, but many other "risk factors" for coronary heat disease[40].

There is no agreement, apart from the statements by various self-appointed committees and the so-called consensus conferences, about what represents "high" blood cholesterol. Cholesterol measurements are notoriously unreliable[41].

According to the sixth King's Fund Consensus Statement, issued on June 28, 1988, "mass measurement of blood cholesterol levels is not justified". This is in direct contradiction to the recommendation by the National Cholesterol Education Programme in the USA, who insist that serum total cholesterol should be measured in all adults 20 years of age and over at least once every 5 years[42].

The Working Group for Cardiovascular Disease of the Faculty of Community Medicine of the Royal College of Physicians of the Royal College of Physicians of the UK, on the other hand, concluded that population screening for cholesterol cannot be justified as it has not been shown that it does not do more harm than good[43]. However, the politics and profits of cholesterol screening have taken over rational discussion[37,38] and there are signs that the cholesterol hysteria which now rages in the USA will infect European countries in the near future.

### CONCLUSION

It is unethical to offer the public screening programmes without honest assessment of the balance of risks and benefits and without providing this information to the prospective participants. Current screening programmes for cancers of the breast and cervix, and for the risk factors for coronary heart disease fail to meet Wilson and Jungner's criteria for rational screening and should be suspended.

### REFERENCES

1. WILSON, J.M.G., JUNGNER, G. (1968) Principles and practice of screening for disease. Geneva: WHO.
2. MCKEOWN, T. (1968) Validation of screening procedures. In: Screening in medical care: reviewing the evidence. London: Nuffield Provincial Hospitals Trust.
3. COCHRANE, A.L., HOLLAND, W.W. (1971) Validation of screening procedures. *Br. Med. Bull.* **27**, 3–8.
4. SACKETT, D., HOLLAND, W.W. (1975) Controversy in the detection of disease. *Lancet*, **II**, 357–359.
5. OLSEN, D.M., KANE, R.L., PROCTOR, P.H. (1976) A controlled trial of multiphasic screening. *N. Engl. J. Med.* **294**, 925–930.
6. HOLLAND, W.W., CREESE, A.L., D'SOUZA, M.F., PARTRIDGE, J.R.J., SHANNON, D., STONE, D.H., SWAN, A.V., TREVELYAN, H.T., TUCKMAN, E., WOODALL, H.T.J. (1977) A controlled trial of multiphasic screening in middle age: results of the South East London Screening Study. *Intern. J. Epidemiol.* **6**, 357–363.
7. SKRABANEK, P. (1985) False premises and false promises of breast cancer screening. *Lancet*, **ii**, 316–319.
8. WRIGHT, C.J. (1986) Breast cancer screening: a different look at the evidence. *Surgery*, **100**, 594–598.
9. SKRABANEK, P. (1989) The debate over mass mammography in Britain: the case against. *Br. Med. J.* **297**, 971–972.
10. SKRABANEK, P. (1989) Mass mammography: the time for reappraisal. *Intern. J. Technol. Assessment Health Care*, **5**, 423–430.
11. SKRABANEK, P. (1989) Shadows over screening mammography (editorial). *Clin. Radiol.* **40**, 4–5.
12. ANDERSSON, I., ASPERGEN, K., JANZON, L., LANDBERG, T., LINDHOLM, K., LINELL, F., LJUNGBERG, O., RANSTAM, J., SIGFUSSON, B. (1988) Effect of mammographic screening on breast cancer mortality in an urban population in Sweden. Results from the randomised Malmö mammographic screening trial. *Br. Med. J.* **297**, 943–948.
13. GULLBERG, B., ANDERSSON, I., JANZON, L., RANSTAM, J. (1991) Screening mammography. *Lancet,* **337,** 244.
14. ROBERTS, M.M., ALEXANDER, F.E., ANDERSON, T.J., CHETTY, U., DONNAN, P.T., FORREST, P., HEPBURN, W., HUGGINS, A., KIRKPATRICK, A.E., LAMB, J., MUIR, B.B., PRESCOTT, R.J. (1990) Edinburgh trial of screening of breast cancer: mortality at seven years. *Lancet*, **335**, 241–246.
15. ROBERTS, M.M. (1989) Breast screening: time for a rethink? *Br. Med. J.*, **299**, 1153–1155.
16. BAINES, C.J., MILLER, A.B., WALL, C., MCFARLANE, D.V., SIMOR, I.S., JONG, R., SHAPIRO, B.J. (1986) Sensitivity and specificity of first screen mammography in the Canadian National Breast Screening Study. *Radiology*, **160**, 295–298.
17. KETCHAM, A.S., MOFFAT, F.L. (1990) Vexed surgeons, perplexed patients and breast cancer which may not be cancer. *Cancer*, **65**, 387–393.
18. SCHMIDT, J. (1990) The epidemiology of mass breast cancer screening — a plea for a valid measure of benefit. *J. Clin. Epidemiol.* **43**, 215–239.
19. KOSS, L.G. (1989) The Papanicolaou test for cervical cancer detection: a triumph and a tragedy. *JAMA*, **261**, 737–743.
20. ISMAIL, S.M., COLCLOUGH, A.B., DINNEN, J.S., EAKINS, D., EVANS, D.M.D., GRADWELL, E., O'SULLIVAN, P., SUMMERELL, J.M., NEWCOMBE, R.G. (1989) Observer variation in histopathological diagnosis and grading of cervical intraepithelial neoplasia. *Br. Med. J.* **298**, 707–710.
21. GILES, J.A., HUDSON, E., CROW, J., WILLIAMS, D., WALKER, P. (1988) Colposcopic assessment of the accuracy of cervical cytology screening. *Br. Med. J.* **296**, 1099–1102.
22. ANON (1985) Cancer of the cervix: death by incompetence (editorial). *Lancet* **ii**: 363–364.
23. ROBIN, E.D. (1985) Failure of the cervical cytology screening programme. *Br. Med. J.* **290**, 75.
24. SKRABANEK, P. (1987) Cervical cancer screening. *Lancet*, **ii**: 510.
25. SKRABANEK, P. (1988) Cervical cancer screening; the time for reappraisal. *Canad. J. Public Health*, **79**, 86–89.
26. MCCORMICK, J.S. (1989) Cervical smears: a questionable practice? *Lancet*, **ii**: 207–209.
27. SKRABANEK, P. (1989) Mass screening in women: more harm than benefit? In: BA Stoll. ed. Social dilemmas in cancer prevention. London: Macmillan. 67–73.
28. CORDAY, E., CORDAY, S.R. (1975) Prevention of heart disease by control of risk factors: the time has come to face the facts. *Am. J. Cardiol.* **35**, 330–333.
29. MANN, G.V. (1977) Diet-heart: end of an era. *N. Engl. J. Med.* **297**, 644–650.
30. BORHANI, N.O. (1977) Primary prevention of coronary heart disease: a critique. *Am. J. Cardiol.*, **40**, 251–259.
31. MITCHELL, J.R.A. (1984) What constitutes evidence on the dietary prevention of coronary heart disease: cosy beliefs or harsh facts? *Intern. J. Cardiol.* **5**, 287–298.
32. AHRENS, E.H. (1985) The diet-heart question in 1985: has it really been settled? *Lancet*, **i**: 1085–1087.

*(Cont'd on page 45)*

### SCREENING FOR DISEASE

West of England Medical Journal Volume 106)ii) June 1991, *Skrabanek*

*Continued from p. 39*

33. WERKÖ, L. (1987) The enigma of coronary heart disease and its prevention. *Acta Med. Scand.* **221**, 323–333.
34. BECKER, M.H. (1987) The cholesterol saga: whither health promotion. *Ann. Intern. Med.* **106**, 623–626.
35. PALUMBO, P.J. (1988) National cholesterol education program: does the emperor have any clothes? *Mayo Clin. Proc.* **63**, 88–90.
36. OLIVER, M.F. (1988) Reducing cholesterol does not reduce mortality. *J. Am. Coll. Cardiol.* **12**, 814–817.
37. SMITH, R.L. (1988) Diet, blood cholesterol and coronary heart disease: a critical review of the literature. Santa Monica, CA: Victor Enterprises.
38. MOORE, T.J. (1989) Heart failure. A critical inquiry into American medicine and the revolution in heart care. New York: Random House.
39. FRANTZ, I.D., DAWSON, E.A., ASHMAN, P.L., GATEWOOD, L.C., BARTSCH, G.E., KUBA, K., BREWER, E.R. (1989) Test of effect of lipid lowering by diet on cardiovascular risk. The Minnesota Coronary Survey. *Arteriosclerosis*, **9**, 129–135.
40. MCCORMICK, J.S., SKRABANEK, P. (1988) Coronary heart disease is not preventable by population interventions. *Lancet*, **ii**: 839–841.
41. Laboratory Standardization Panel of the National Cholesterol Education Program. (1988) Current status of blood cholesterol measurement in clinical laboratories in the United States. *Clin. Chem.* **34**, 193–201.
42. THE EXPERT PANEL. (1988) Report of the National Cholesterol Education Program Expert Panel on detection, evaluation, and treatment of high blood cholesterol in adults. *Arch. Intern. Med.*, **148**, 36–69.
43. SMITH, W.C.S., KENICER, M.B., DAVIS, A.M., EVANS, A.E., YARNELL, J. (1989) J. Blood cholesterol: is population screening warranted in the UK? *Lancet*, **i**: 372–373.

